# Characteristics of androgen metabolic pathways in patients with 21-hydroxylase deficiency and their association with disease control status

**DOI:** 10.3389/fendo.2026.1762815

**Published:** 2026-05-11

**Authors:** Hemeng Chong, Yalei Pi, Yanan Zhang, Yuqian Li, Yutong Xing, Huifeng Zhang

**Affiliations:** Department of Pediatrics, The Second Hospital of Hebei Medical University, Shijiazhuang, Hebei, China

**Keywords:** 21-hydroxylase deficiency, androgen metabolic pathways, classical pathway dominance, disease control status, puberty

## Abstract

**Objective:**

To investigate the activity characteristics of distinct androgen metabolic pathways in patients with 21-hydroxylase deficiency (21OHD) and their correlation with disease control status, and to elucidate the metabolic pathway features and underlying mechanisms in poorly controlled patients.

**Methods:**

A total of 111 patients with confirmed 21OHD were enrolled in this study. Clinical data and steroid hormone profiles were collected, and robust standardized Z-scores were calculated for each steroid hormone. K-means clustering analysis was performed using the robust standardized Z-scores of 7 signature steroid hormones to stratify patients into a well-controlled group and a poorly controlled group. Differences in clinical characteristics, pathway activities and key enzyme conversion efficiencies were compared between and within the two groups.

**Results:**

K-means clustering analysis classified 102 patients into the well-controlled group and 9 into the poorly controlled group. Multivariate analysis identified puberty as an independent risk factor for poor disease control (OR = 11.90, 95%CI:1.43–98.79, P = 0.02). Among pubertal patients, the poorly controlled group had a significantly higher relative weight of classical pathway androgen load than the well-controlled group (63.92% vs. 46.19%, P = 0.02), and the proportion of the classical pathway in the poorly controlled group was markedly higher than that of the 11-oxygenated and backdoor pathways (both P<0.05). Additionally, the conversion efficiency of the 21-deoxycortisol (21DOF) pathway was significantly decreased in the poorly controlled group (P = 0.004), suggesting relatively restricted CYP11B1 activity which drives the shift of androgen metabolism toward the classical pathway.

**Conclusion:**

In poorly controlled pubertal patients with 21OHD, the relative insufficiency of CYP11B1 activity and the dysregulation of the metabolic network jointly lead to the shift of androgen synthesis to the classical pathway, forming a “classical pathway dominance”. This finding deepens the understanding of the pathological mechanism of 21OHD from the perspective of pathway dynamics and provides a novel theoretical basis for individualized clinical treatment of the disease.

## Introduction

21-hydroxylase deficiency (21OHD) is the most common form of congenital adrenal hyperplasia (CAH), characterized by impaired cortisol synthesis and excessive androgen production. Androgen synthesis in 21OHD patients is mainly mediated through three metabolic pathways: the classical pathway, the 11-oxygenated androgen pathway, and the backdoor pathway. Chronic elevation of adrenocorticotropic hormone (ACTH) can enhance the activity of CYP17A1, promoting the conversion of 17-hydroxyprogesterone (17OHP) to androstenedione (AD) and thus upregulating androgen synthesis via the classical pathway. Increased CYP11B1 activity leads to the massive production of 11β-hydroxyandrostenedione (11OHAD), whose concentration is higher than that of AD in both 21OHD patients and healthy controls (1). 11OHAD can be further converted to 11-ketotestosterone (11KT) by 11β-hydroxysteroid dehydrogenase type 2 and AKR1C3, with biological activity similar to testosterone (T) (2). Progesterone or 17OHP can also synthesize highly active dihydrotestosterone (DHT) through the backdoor pathway, in which androsterone (Andr) is a key intermediate. Metabolites of this pathway can be detected in 21OHD patients of all ages, especially in neonates, indicating that high levels of 17OHP can drive androgen synthesis via the backdoor pathway (3). Furthermore, in the absence of 21-hydroxylase activity, a portion of 17OHP is converted to 21-deoxycortisol (21DOF) by CYP11B1, which has been identified as a potential diagnostic biomarker for 21OHD (4).

Currently, the monitoring of steroid hormones in 21OHD still focuses on 17OHP and AD, the classic markers of the classical pathway, while the clinical value of adrenal-specific 11-oxygenated androgens and 21DOF in disease treatment monitoring remains controversial. In recent years, metabonomic studies combining urinary steroidomics and cluster analysis have emerged as a new direction for 21OHD treatment monitoring. Detection of urinary steroid biomarkers by gas chromatography-mass spectrometry (GC-MS) and liquid chromatography-tandem mass spectrometry (LC-MS/MS) can effectively classify the disease control status of pediatric and infant patients (5–7), and the introduction of machine learning has further improved the predictive efficacy of treatment outcomes (8). However, these studies all use urine as samples and focus on young children, without conducting analyses for puberty—a critical stage for disease control—nor in-depth dissection of the activity characteristics of specific androgen metabolic pathways. Based on this, the present study systematically analyzed the activity of androgen metabolic pathways in 21OHD patients characterized by plasma steroids, explored their association with disease control status, and provided a reference for precise diagnosis and treatment of 21OHD.

## Materials and methods

### Study subjects

A total of 111 patients with 21OHD confirmed by clinical phenotypes combined with genetic testing were enrolled in this study.

Inclusion criteria: ①Diagnosis of 21OHD confirmed by clinical phenotypes and genetic testing; ②Regular treatment and follow-up every 3~6 months with complete clinical data and steroid hormone detection results; ③Written informed consent signed by the patients’ guardians.

Exclusion criteria: ①Complicated with other organic diseases of the adrenal gland and gonads; ②Recent use of non-therapeutic drugs that may affect steroid metabolism; ③Loss to follow-up or missing core data during the follow-up period; ④Complicated with severe hepatic and renal insufficiency.

This study was approved by the Ethics Committee of the Second Hospital of Hebei Medical University (approval number: 2024-R563), and all research procedures were in accordance with the relevant requirements of the Declaration of Helsinki.

### Collection of data and specimens

The latest follow-up clinical and laboratory data of the patients were collected, including basic demographic information (age, gender), physical indicators (height, weight, BMI), clinical phenotypes, pubertal status, follow-up duration, and treatment regimens. Pubertal status was independently assessed by two senior pediatric endocrinologists: the onset of puberty in females was defined as Tanner II breast development, and in males as testicular volume ≥4 mL. Discrepancies between the two physicians were resolved through joint discussion.

Fasting peripheral venous blood was collected from patients at 8:00 a.m. without medication and. The blood samples were centrifuged at 3000 r/min for 10 min at 4 °C within 30 min after collection. Plasma steroid hormone levels were detected by LC-MS/MS (Waters-Xevo TQ-S instrument). The detected indicators included 17OHP, 21DOF, AD, T, DHT, Andr, 11OHAD and 11KT, among which 17OHP and AD were markers of the classical pathway, and 11OHAD and 11KT were markers of the 11-oxygenated androgen pathway, Andr was a marker of the backdoor pathway.

### Data processing and definition

SDS score calculation: The standard deviation scores (SDS) of height, weight and BMI were calculated with reference to the *Chinese Reference Standards for Growth and Development of Children and Adolescents (2021 Edition)*, using the formula: SDS=(measured value-median of children of the same age and gender)/standard deviation of children of the same age and gender.

Robust standardized Z-score calculation: Due to the non-normal distribution and inconsistent dimensions of steroid hormones, the robust standardization method was used to standardize all hormone indicators, with the formula: Z=(individual value-overall median)/interquartile range, which was applied for subsequent cluster analysis and pathway activity assessment.

Comprehensive Z-score of metabolic pathways and relative weight of androgen load: The comprehensive Z-score of the classical pathway=(Z-17OHP+Z-AD)/2; the comprehensive Z-score of the 11-oxygenated pathway=(Z-11OHAD+Z-11KT)/2; the comprehensive Z-score of the backdoor pathway=Z-Andr. The total robust Z-score was the sum of the Z-scores of the three pathways, reflecting the overall activity of the androgen pathway. After minimum translation (to eliminate the influence of negative values), the ratio of the comprehensive Z-score of each pathway to the total Z-score was calculated and defined as the relative weight of androgen load, characterizing the relative contribution of each pathway to the androgen pool.

Calculation of key enzyme conversion efficiency: Steroid hormone concentrations were first median-standardized (prefixed with “ms”), and then the conversion efficiency of key enzymes in each pathway was calculated with the following specific indicators:

- Classical pathway: C-E1=msAD/ms17OHP, C-E2=msT/msAD, overall efficiency C-Et=(msT+ msAD)/(2×ms17OHP);
- 11-oxygenated pathway: 11O-E3=ms11OHAD/msAD, 11O-E4=ms11KT/ms11OHAD, overall efficiency 11O-Et=(ms11KT + ms11OHAD)/(2×msAD);
- Backdoor pathway: Bd-E=msAndr/ms17OHP;
- 21DOF pathway: 21DF-E=ms21DOF/ms17OHP;
- CYP11B1 relative activity: 11O-E3/C-E1 and 11O-E3/C-Et reflected the relative enzymatic activity of CYP11B1 involved in the 11-oxygenated androgen pathway; 21DF-E/C-E1 and 21DF-E/C-Et reflected the relative enzymatic activity of CYP11B1 involved in the 21DOF pathway.

### Cluster analysis

IBM SPSS Statistics 26.0 software and R software (stats package and factoextra package) were used for K-means clustering analysis to cross-validate the stability of the results. The clustering indicators were the robust standardized Z-scores of 7 signature steroid hormones (17OHP, AD, T, DHT, Andr, 11OHAD, 11KT), and the optimal number of clusters (k=2) was determined by the elbow method combined with clinical needs. The algorithm parameters were set as Euclidean distance measurement, and the clustering process was independently run 3 times with each software. The clustering results of the two tools were completely consistent, and the patients were finally divided into a well-controlled group (n=102) and a poorly controlled group (n=9), ensuring the reliability of the grouping results.

### Statistical analysis

Quantitative data with non-normal distribution were expressed as M(P25,P75). The Mann–Whitney U test was used for inter-group comparison, and the χ² test for comparison of categorical data. Friedman test was used for intra-group comparisons, and Wilcoxon signed-rank test with Bonferroni correction for pairwise comparisons. Spearman rank correlation analysis was used for correlation analysis. Multivariate Logistic regression analysis was applied to screen for independent risk factors for poor disease control in 21OHD patients. A P-value < 0.05 was considered statistically significant.

## Results

### Effective stratification of 21OHD patients by K-means clustering analysis based on disease control status

Among the 111 enrolled 21OHD patients, 62 were male and 49 were female; 72 had the salt-wasting type (SW), 27 the simple virilizing type (SV), and 12 the non-classical type (NC); 62 were prepubertal and 49 were pubertal.

**Figure 1:**
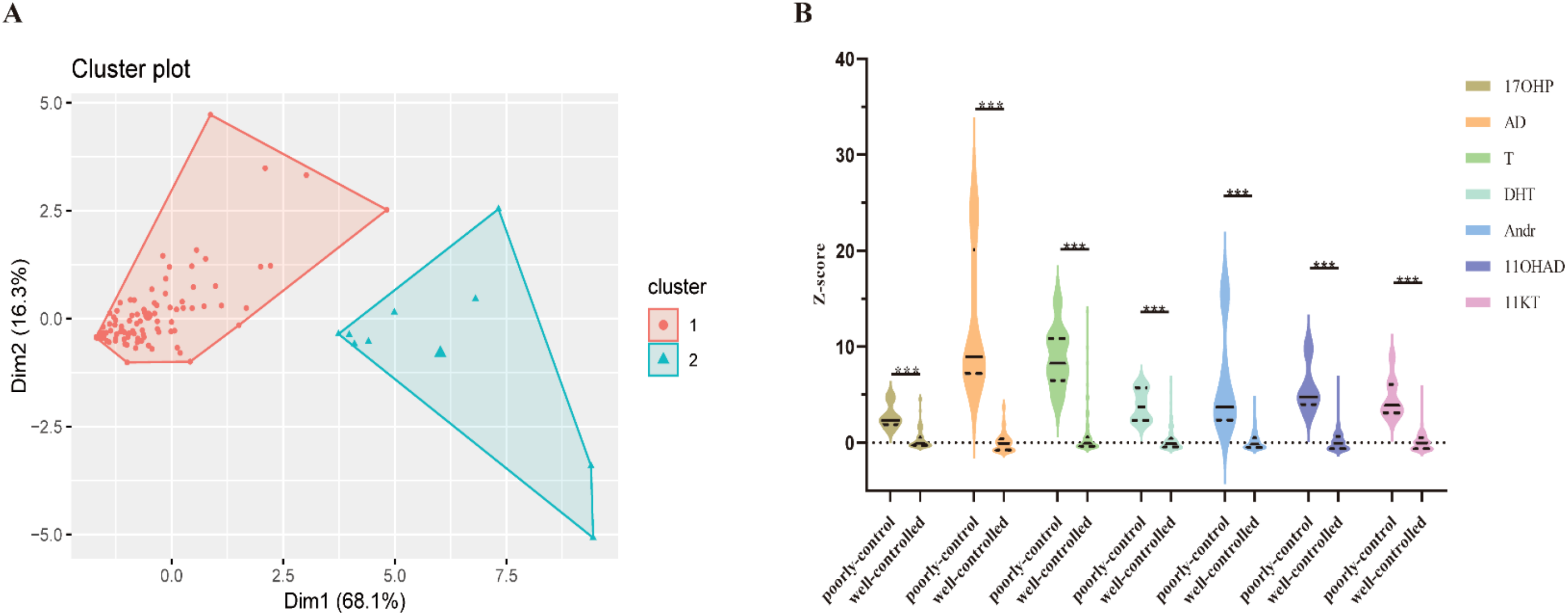
K-means clustering analysis results of 111 21OHD patients based on robust standardized Z-scores of 7 steroid hormones. **(A)** Scatter points represent individual patients. K-means clustering analysis (k=2) was performed based on the robust standardized Z-scores of 7 signature steroid hormones, dividing patients into the well-controlled group (red scatter points, n=102) and the poorly controlled group (blue scatter points, n=9). Dim1 and Dim2 are the two principal component dimensions of the clustering analysis, with the variance explained by each dimension shown in parentheses (Dim1 = 68.1%, Dim2 = 16.3%). A significant separation trend in hormone levels was observed between the two groups. **(B)**. Extremely significant differences were found in the robust standardized Z-scores of all 7 signature steroid hormones between the well-controlled and poorly controlled groups (all P<0.001). *** indicates statistical significance (P < 0.001).

K-means clustering analysis divided all patients into two subgroups (**Figure 1A**): the well-controlled group (n=102) with generally low steroid hormone levels, and the poorly controlled group (n=9) with elevated levels of all steroid hormones across the spectrum. There were extremely significant differences in the robust standardized Z-scores of all 7 signature steroid hormones between the two groups (**Figure 1B**; **Supplementary Table 1**, all P<0.001).

### Puberty as an independent risk factor for poor disease control in 21OHD patients

Univariate analysis showed that there were statistically significant differences in the proportion of pubertal patients, follow-up duration, age, weight SDS and BMI-SDS between the poorly controlled and well-controlled groups (all P<0.05, **Table 1**). Variables with P<0.05 in the univariate analysis were included in the multivariate Logistic regression analysis, and only pubertal status entered the final regression model, confirming that puberty was an independent risk factor for poor disease control in 21OHD patients (OR = 11.90, 95%CI:1.4398.79, P = 0.02), To further investigate the core molecular mechanisms underlying poor disease control, subsequent analyses focused specifically on pubertal patients.

**Table 1:** Analysis of baseline characteristics and influencing factors of disease control status in 111 21OHD patients.

Characteristic	Univariate analysis				Multivariate logistic regression analysis			
	Poorly-controlled group (n=9)	Well-controlled group (n=102)	Z/χ2	P	b	Wald	P	OR(95%CI)
Gender (male: female)	6:3	56:46	0.46	0.73				
Age(years)	10.92(7.79,12.55)	7.03(3.72,10.26)	2.16	0.03				
Clinical phenotype (SW: SV: NC)	6:2:1	66:25:11	0.25	>0.999				
Pubertal (Yes: No)	8:1	41:61	7.95	0.01	2.48	5.26	0.02	11.90(1.43-98.79)
Follow-up duration	9.24(4.24,12.34)	4.10(1.97,7.52)	2.20	0.03				
Height SDS	0.60(-1.35,2.00)	0.40(-0.70,1.40)	0.28	0.78				
Weight SDS	2.00(0.55,3.50)	0.80(-0.10,1.50)	2.36	0.02				
BMI-SDS	2.50(0.70,2.60)	0.65(0.10,1.83)	2.16	0.03				
Hydrocortisone doses (mg/m^2^)	14.71(9.44,18.47)	12.81(10.08,16.11)	0.65	0.52				
Fludrocortisone dose (mg/d)	0.10(0.00,0.10)	0.05(0.00,0.08)	0.76	0.45				

Univariate analysis: The Mann–Whitney U test was used for quantitative data, and the χ² test for comparison of categorical data. Multivariate Logistic regression analysis adopted the forward stepwise method (LR method), including and screening variables with P<0.05 in univariate analysis, and only pubertal status was finally included; OR=Odds Ratio, 95%CI=95% Confidence Interval.

### Specifically increased relative weight of classical pathway androgen load in poorly controlled pubertal patients

**Figure 2:**
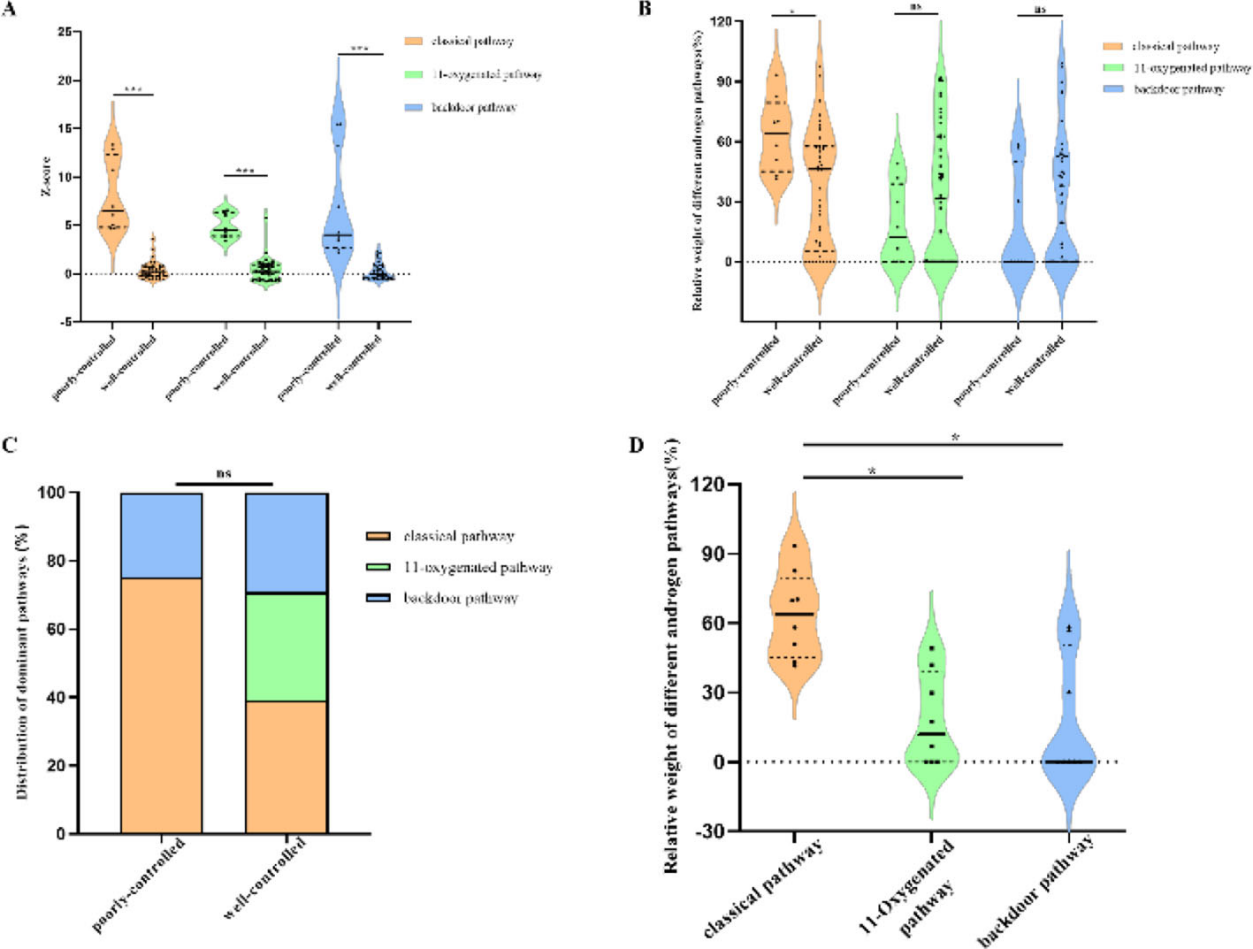
Characteristics of androgen metabolic pathway activity, load weight and dominant pathway distribution in pubertal 21OHD patients. **(A)** Comparison of robust Z-scores of the classical, 11-oxygenated and backdoor pathways between the poorly controlled and well-controlled groups; **(B)** Difference in the relative weight of classical pathway androgen load between the two groups; **(C)** Distribution ratio of dominant metabolic pathways between the two groups; **(D)** Pairwise comparison results of relative weights of androgen load among the three pathways in the poorly controlled group. *P < 0.05, *** P < 0.001.

Among pubertal patients, there were no statistically significant differences in baseline data between the poorly controlled group (n=8) and the well-controlled group (n=41) (**Supplementary Table 2**, all P>0.05), ensuring the comparability between the two groups. Analysis of steroid metabolic pathways showed that the robust Z-scores of the classical, 11-oxygenated and backdoor pathways in the poorly controlled group were significantly higher than those in the well-controlled group (**Figure 2A**; **Supplementary Table 3**, all P<0.001). Among them, the relative weight of classical pathway androgen load in the poorly controlled group was significantly higher than that in the well-controlled group (**Figure 2B**; **Supplementary Table 3**, median 63.92% vs. 46.19%, P = 0.02). Analysis of dominant pathway distribution showed that the proportion of patients with different dominant metabolic pathways was relatively balanced in the well-controlled group, while the proportion of patients with classical pathway dominance was higher in the poorly controlled group (75.00% vs. 39.02%), with no statistically significant difference in the composition ratio of dominant pathways between the two groups (**Figure 2C**; **Supplementary Table 3**, P = 0.14).

Intra-group pairwise comparisons showed no statistically significant difference in the distribution of relative weights of androgen load among the three pathways in the well-controlled group (P = 0.39), while the relative weight of classical pathway androgen load in the poorly controlled group was significantly higher than that of the 11-oxygenated and backdoor pathways (**Figure 2D**; **Supplementary Table 3**, median 63.92% vs. 12.07% and 0.00%, both P<0.05). Multivariate Logistic regression analysis further confirmed that a higher relative weight of classical pathway androgen load was associated with an increased risk of poor disease control in pubertal 21OHD patients (OR = 1.039, 95%CI:1.003–1.076, P = 0.03).

### Absolute dominance of classical pathway enzyme conversion efficiency in poorly controlled pubertal patients

Inter-group comparison of key enzyme conversion efficiencies of each metabolic pathway in pubertal patients showed (**Table 2**) that the conversion efficiency of the 21DOF pathway (21DF-E) in the poorly controlled group was significantly lower than that in the well-controlled group (median 0.34 vs. 0.75, P = 0.004), while there were no statistically significant differences in the enzyme conversion efficiencies of other pathways between the two groups, suggesting that the CYP11B1-mediated conversion of 17OHP to 21DOF was impaired, a characteristic enzymatic activity specific to poorly controlled pubertal patients.

**Table 2:** Inter-group and Intra-group comparison of key enzyme conversion efficiencies of each metabolic pathway in pubertal 21OHD patients [M(P25,P75)].

Index	Poorly-controlled group (n=8)	Well-controlled group (n=41)	Z	P
C-E1	0.90(0.85,2.03)a	1.13(0.37,3.75)	0.18	0.86
C-E2	0.94(0.62,1.38)ab	0.82(0.58,1.47)	0.49	0.63
C-Et	0.94(0.81,1.69)a	1.21(0.33,4.55)	0.46	0.65
11O-E3	0.45 (0.38,0.56)	0.71(0.36,0.96)	1.85	0.06
11O-E4	0.46(0.19,0.63)	0.69(0.34,1.06)	1.73	0.08
11O-Et	0.50(0.27,0.60)	0.68(0.38,1.03)	1.79	0.07
Bd-E	0.69(0.52,2.13)	0.86(0.31,2.04)	0.22	0.83
21DF-E	0.34(0.20,0.45)	0.75(0.43,1.61)	2.91	0.004

Inter-group comparison: The Mann–Whitney U test was used to compare the conversion efficiency of each androgen metabolic pathway enzyme between the poorly-controlled group and well-controlled group in adolescents. Intra-group comparison: Friedman test revealed no significant difference in enzyme conversion efficiency among metabolic pathways in the well-controlled pubertal group (χ²=8.35, P = 0.30), but a significant difference in the poorly-controlled group (χ²=30.64, P<0.001). Wilcoxon signed-rank tests with Bonferroni correction showed significant differences between the classic pathway (C-E1, C-E2, C-Et) and 21DOF pathway (21DF-E1) (a), and between the classic pathway (C-E2) and 11-oxygenated pathway (11O-E4) (b) in the poorly-controlled group.

There was no statistically significant difference in the conversion efficiency of key enzymes across each metabolic pathway in the well-controlled pubertal group (**Table 2**, P > 0.05). In the poorly-controlled pubertal group, however, comparison of enzyme conversion efficiencies (**Table 2**) revealed that the classical pathway exhibited significantly higher efficiency than both the 11-oxygenated pathway and the 21DOF pathway (all P < 0.05). This suggests that the enzyme conversion efficiency of the classical pathway occupied an absolute advantage in the androgen metabolic network of poorly controlled pubertal patients.

### Significantly dysregulated correlation pattern of androgen metabolic pathway enzyme conversion efficiency in poorly controlled pubertal patients

**Figure 3:**
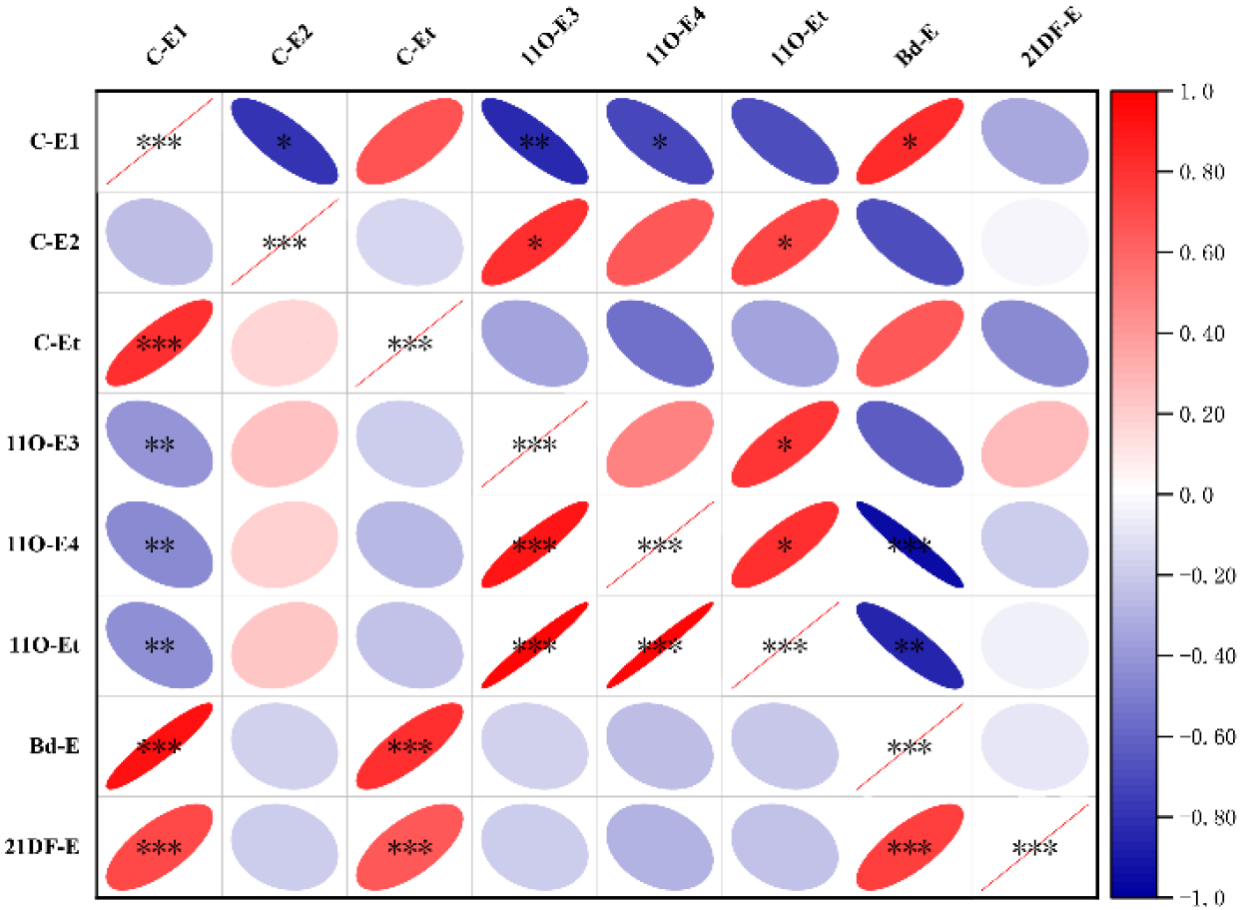
Correlation heatmap of key enzyme conversion efficiencies of each metabolic pathway in pubertal 21OHD patients. The shade of color in the heatmap represents the value of Spearman correlation coefficient r (red for positive correlation, blue for negative correlation). The left side is the well-controlled group and the right side is the poorly controlled group; rows and columns are all key enzyme conversion efficiency indicators of each metabolic pathway. The metabolic network of the well-controlled group shows coordinated correlation characteristics, while the negative correlation between various pathways and within the classical pathway is enhanced in the poorly controlled group, suggesting metabolic network dysregulation. *P<0.05, **P<0.01, ***P<0.001.

Correlation analysis of key enzyme conversion efficiencies of androgen metabolic pathways showed significant differences in correlation patterns between the two groups (**Figure 3**; **Supplementary Table 4**). In the well-controlled group, the key enzyme of the classical pathway (C-E1) was weakly negatively correlated with the related enzymes of the 11-oxygenated androgen pathway (*r* = -0.45 to -0.41, P<0.01); the classical pathway was moderately to strongly positively correlated with the backdoor pathway and the 21DOF pathway, and the backdoor pathway was also moderately to strongly positively correlated with the 21DOF pathway (*r* = 0.66 to 0.92, P<0.01), showing a coordinated correlation characteristic of the overall metabolic network.

In the poorly controlled group, the correlation pattern of the metabolic pathway was significantly disordered: a strong negative correlation was observed within the classical pathway (C-E1 and C-E2, *r* = -0.79, P<0.05); C-E1 was moderately to strongly negatively correlated with the key enzyme conversion efficiencies of the 11-oxygenated pathway (*r* = -0.71 to -0.84, P<0.05); a strong negative correlation existed between the 11-oxygenated and backdoor pathways (*r* = -0.95, P<0.001); only a strong positive correlation was maintained between the classical and backdoor pathways (*r* = 0.83, P<0.05). The above results indicated that compared with the well-controlled group, the enzyme conversion efficiencies in the poorly controlled group showed stronger negative correlations between various pathways and within the classical pathway, suggesting that the coordination of its androgen metabolic network was severely impaired, presenting a significant characteristic of metabolic network dysregulation.

### Relatively low catalytic efficiency of CYP11B1 for the 21DOF pathway in poorly controlled pubertal patients

Comparison of the relative activity of CYP11B1 and the classical pathway between the two groups showed (**Table 3**) that 11O-E3/C-E1 (median 0.51 vs. 0.80, P = 0.68) and 11O-E3/C-Et (median 0.44 vs. 0.46, P = 0.77), which reflected the relative activity of CYP11B1 involved in the 11-oxygenated androgen metabolic pathway, had no statistically significant differences between the two groups; while 21DF-E/C-E1 (median 0.31 vs. 0.95, P = 0.02) and 21DF-E/C-Et (median 0.25 vs. 0.88, P = 0.01), which reflected the relative activity of CYP11B1 involved in the 21DOF pathway, were significantly lower in the poorly controlled group than in the well-controlled group. The results suggested that the catalytic efficiency of CYP11B1 for converting 17OHP to 21DOF was relatively lower in poorly controlled pubertal patients, and the enzyme had a significantly higher affinity for substrate AD than for 17OHP.

**Table 3:** Inter-group comparison of relative CYP11B1 activity in pubertal 21OHD patients [M(P25,P75)].

Index	Poorly-controlled groups	Well-controlled groups	Z	P
11O-E3/C-Et	0.44(0.26,0.60)	0.46(0.08,1.59)	0.30	0.77
11O-E3/C-E1	0.51(0.21,0.63)	0.80 (0.08,1.69)	0.42	0.68
21DF-E/C-E1	0.31(0.16,0.44)	0.95 (0.44,1.78)	2.42	0.02
21DF-E/C-Et	0.25(0.18,0.51)	0.88 (0.37,1.49)	2.68	0.01

The Mann–Whitney U test was used to compare the relative CYP11B1 activity in pubertal 21OHD patients .

## Discussion

Through multidimensional analysis of clinical data and steroid hormone profiles of 111 21OHD patients, this study identified puberty as an independent risk factor for poor disease control, and further clarified the core characteristic of “classical pathway dominance” in androgen metabolism of poorly controlled pubertal patients, while elucidating the molecular mechanism by which the relative insufficiency of CYP11B1 activity and metabolic network dysregulation jointly drive the formation of this characteristic, providing a new theoretical basis for the individualized treatment of 21OHD.

We use K-means clustering analysis to stratify the control status of 21OHD patients based on the robust standardized Z-scores of plasma steroids, a method whose effectiveness has been verified in multiple CAH urinary steroid metabonomic studies (5, 6). Compared with previous urine sample clustering studies (5–8), the robust standardization method adopted in this study effectively eliminates the influence of non-normal distribution and inconsistent dimensions, more accurately evaluates the relative activity of various steroid hormones and metabolic pathways, and better reflects the metabolic differences within the 21OHD patient group, which is also an important methodological basis for this study to deeply reveal the characteristics of pathway dynamics. The results showed that the robust Z-scores of the three androgen metabolic pathways were significantly increased in the poorly controlled group, indicating that such patients had not a specific abnormality of a single metabolic pathway, but a systemic and multi-pathway upregulation of androgen synthesis pathways. This research suggests that in the clinical management of refractory 21OHD patients, attention should be paid to the inhibition of the overall androgen load, rather than only focusing on the regulation of a single hormone level; the pathway robust Z-score can be used as a comprehensive index to evaluate the overall disease activity of 21OHD, and the relative weight of androgen load can be used as an important reference index to distinguish the disease control status of patients.

In clinical practice, it is common that pubertal patients still present with poorly controlled androgen levels despite adequate glucocorticoid dosages and good treatment adherence, which is highly consistent with the finding of this study that puberty serves as an independent risk factor for suboptimal disease control in 21OHD patients. The core pathological mechanism lies in abnormal cortisol pharmacokinetics induced by the altered endocrine milieu during puberty, characterized by a marked increase in cortisol clearance and volume of distribution (9). Firstly, elevated sex steroids in puberty upregulate the activity of the growth hormone-insulin-like growth factor I (GH-IGF-I) axis: on the one hand, it inhibits the activity of hepatic 11β-hydroxysteroid dehydrogenase type 1 (11β-HSD1), reducing the conversion of cortisone to cortisol and accelerating the metabolic clearance of cortisol (10–13); on the other hand, it elevates the glomerular filtration rate, thereby further increasing the renal clearance of cortisol (14, 15). Secondly, the increased body surface area and altered tissue binding characteristics during puberty lead to a significant rise in the volume of cortisol distribution (9). The synchronous elevation of cortisol clearance and volume of distribution makes it difficult to maintain the effective plasma concentration of exogenous hydrocortisone replacement therapy, which results in insufficient suppression of the hypothalamic-pituitary-adrenal axis and excessive secretion of ACTH. In addition, IGF-I and insulin can directly promote the expression of steroidogenic enzymes in the adrenal cortex (16, 17), which together facilitate excessive synthesis of adrenal androgens and ultimately lead to suboptimal disease control in pubertal patients.

The discovery of “classical pathway dominance” in poorly controlled pubertal patients reveals the fundamental remodeling of their intrinsic metabolic pattern, which is consistent with the results of multiple urinary steroidomics studies in 21OHD patients that insufficiently treated patients generally have accumulation of 17OHP and androgen metabolites (5–7). However, some studies (1, 18–20) failed to distinguish the disease control status of 21OHD patients, the metabolic characteristic of classical pathway dominance was masked, and only the adrenal-specific 11-oxygenated and backdoor pathways were observed to be more prominent than those in healthy people. This study further disassembles the specific androgen metabolic pathways and finds that the relative weight distribution of the three androgen metabolic pathways is relatively balanced in the well-controlled group, suggesting that its metabolic network is in a compensatory steady state; while the metabolic network in the poorly controlled group is significantly inclined to the classical pathway, showing the characteristic of “single pathway dominance”. This transition from “multi-pathway synergistic compensation” to “single pathway abnormal dominance” is an important pathophysiological turning point for 21OHD patients from disease compensation to decompensation, providing a new perspective for understanding the disease progression mechanism.

The formation of “classical pathway dominance” is not caused by a single factor, but a self-reinforcing metabolic cycle formed by the synergistic amplification and interaction of multiple mechanisms. First is the reprogramming of androgen metabolic enzyme conversion efficiency: the overall conversion efficiency of the classical pathway in the poorly controlled group has a significant advantage over the 11-oxygenated pathway, becoming the original driving force for the production of a large number of active androgens. Second is the relative insufficiency of CYP11B1 activity and substrate preference: the production of 11OHAD and 21DOF both depends on CYP11B1, but AD is a good substrate for CYP11B1 (1) with higher affinity than 17OHP. This substrate preference leads to a key metabolic shunt: a large amount of AD produced by the classical pathway is preferentially used for the synthesis of 11-oxygenated androgens, which relatively weakens the pathway of 17OHP synthesizing 21DOF. This mechanism not only explains the phenomenon that the precursor production of the 11-oxygenated pathway is not reduced in poorly controlled patients, but more importantly, leads to the massive accumulation of 17OHP, a key intermediate product, which is forced to “spill over” to the classical and backdoor pathways, further aggravating the self-reinforcement of the classical pathway and the abnormal activation of the backdoor pathway. Third is the overall dysregulation of the metabolic network: correlation analysis of enzyme activities of each metabolic pathway reveals that the poorly controlled group shows a stronger negative correlation between various pathways, reflecting the damage of metabolic network coordination and the intensification of competition for common substrates between pathways. In such a metabolic environment, the classical pathway, which already has an efficiency advantage and obtains substrate supplementation, finally “wins” and occupies a dominant position, forming the metabolic characteristic of “classical pathway dominance”.

The identification of the metabolic characteristic of “classical pathway dominance” in this study provides a new direction and theoretical support for the clinical treatment of 21OHD, suggesting that inhibiting ACTH release or specifically interfering with androgen synthesis via the classical pathway may become a key strategy to improve the treatment effect of poorly controlled pubertal patients. The combination of Corticotropin-Releasing Hormone (CRH) and its type 1 receptor (CRF1R) is the main factor stimulating ACTH secretion. Studies have confirmed that CRF1R antagonists can reduce the levels of steroid hormones such as ACTH, 17OHP, T and AD to clinically significant levels (21, 22). Modified-release hydrocortisone can significantly improve the levels of 17OHP and AD in 21OHD patients by optimizing the administration mode of glucocorticoids, and have a positive impact on the long-term fertility-related indicators of patients (23). In addition, potent CYP117A1 inhibitors can reduce AD and T in 21OHD patients (24) and also effectively inhibit the synthesis of 11-oxygenated androgens (25). Although there is little application data of the above treatment regimens in pubertal patients with poor conventional treatment, the characteristic of “classical pathway dominance” found in this study provides an important theoretical basis for such patients to receive more active targeted intervention treatment, and also points out the direction for the development of subsequent relevant clinical studies.

*In vitro* experiments show that the affinity of steroid hormones such as 21DOF, 17OHP and progesterone for glucocorticoid receptors is between 24% and 43% of that of cortisol, among which the transcriptional activity of 21DOF is between 8.5% and 17% of that of cortisol, significantly higher than that of 17OHP and progesterone (their transcriptional activities are 0.2% and 0.8% respectively) (26, 27). The results suggest that in the case of 21-hydroxylase deficiency, the conversion process of 17OHP to 21DOF may have a certain endogenous glucocorticoid-like activity, thus playing a partial compensatory role to make up for the defect of insufficient cortisol synthesis. This study found that the conversion efficiency of the 21DOF pathway in poorly controlled pubertal patients was significantly reduced, and the ratio of its relative activity to the classical pathway was also significantly decreased, reflecting that the ability of CYP11B1 to convert 17OHP to 21DOF was relatively insufficient in such patients. Combined with the above *in vitro* experimental results, it can be speculated that when the level of 17OHP is elevated in 21OHD patients, if 21DOF fails to increase correspondingly, it suggests that the activity of CYP11B1 is relatively limited, and its endogenous glucocorticoid-like compensatory effect is insufficient, which ultimately leads to worse overall disease control status of patients. This speculation further clarifies the role of insufficient CYP11B1 activity in the progression of 21OHD, and also provides a theoretical reference for clinical evaluation of patients’ compensatory status by monitoring 21DOF levels.

This study still has certain limitations. First, in the cluster analysis, patients with generally low hormone levels were classified into the well-controlled group, without subdividing into the appropriate treatment group and over-treatment group as in urinary steroid metabonomic studies (5, 6). Second, the sample size of this study is limited, especially in the poorly controlled group, which may affect the statistical power of some tests, but non-parametric tests were adopted throughout the study, which have stronger adaptability to small sample data. Third, limited by the sample size of rare diseases, this study cannot carry out stratified analysis of early and late puberty, which is an objective limitation of this study; however, this study analyzes puberty as a whole, which has fully clarified its pathophysiological mechanism as an independent risk factor for poor 21OHD control, and the study data show no staging-related confounding bias, so this limitation does not affect the scientificity and reliability of the study conclusions. Fourth, this study selected androsterone as the biomarker of the backdoor pathway, while androsterone is also a metabolite of classical pathway androgens (28), which may overestimate the absolute activity of the backdoor pathway to a certain extent, but the robust standardization method adopted in this study evaluates the relative contribution of pathways based on inter-patient comparison in the cohort, which has greatly reduced the influence of this confounding factor. Fifth, this study only analyzes the characteristics of hormone metabolic pathways at the detection node, nor conducting a correlation analysis between this biochemical grouping and clinical hyperandrogenic phenotypes, lacking long-term follow-up data of hormone levels and clinical phenotypes, and cannot reveal the dynamic change law of androgen metabolic pathway characteristics and its association with disease prognosis. Follow-up studies need to carry out multi-center prospective studies, combine steroid detection of plasma and 24h urine samples, conduct more refined disease control status classification of patients, and verify the predictive value of androgen metabolic pathway characteristics combined with clinical phenotypes and long-term follow-up data to screen non-invasive and specific monitoring indicators.

## Conclusion

In summary, this study concludes that in poorly controlled pubertal patients with 21OHD, the relative limitation of CYP11B1 activity and the overall dysregulation of the androgen metabolic network form a synergistic effect, which jointly drives the precursor substances to be metabolized preferentially through the classical pathway with higher conversion efficiency, and ultimately leads to a significant increase in the relative weight of the classical pathway in their androgen pool, forming the metabolic characteristic of “classical pathway dominance”. This finding goes beyond the traditional single hormone level analysis, deepens the understanding of the pathological mechanism of 21OHD from the perspective of pathway dynamics, not only provides new indicators for disease stratification and condition assessment of 21OHD patients, but also provides a new direction and theoretical basis for the precise and individualized treatment of poorly controlled pubertal patients, which is of great significance for promoting the development of clinical diagnosis and treatment of 21OHD.

## Data Availability

The original contributions presented in the study are included in the article/**Supplementary Material**. Further inquiries can be directed to the corresponding author.
