# Supplementary material for: Characteristics of androgen metabolic pathways in patients with 21-hydroxylase deficiency and their association with disease control status
Source: Front Endocrinol (Lausanne). 2026 May 11;17:1762815. doi: 10.3389/fendo.2026.1762815 (PMC13199108; doi:10.3389/fendo.2026.1762815)
Supplement: Supplementary file 1 [file Table1.docx]

| **Supplementary Table** 1: Final cluster centers and results of the F-test from the K-means clustering analysis | | | | | |
| --- | --- | --- | --- | --- | --- |
|  | Final cluster centers | |  | F-test | |
|  | well-controlled group | poorly-controlled group |  | F | P |
| Z_17OHP | 0.34 | 2.73 |  | 48.50 | <0.001 |
| Z_AD | 0.01 | 12.53 |  | 274.60 | <0.001 |
| Z_Andr | 0.17 | 6.26 |  | 104.82 | <0.001 |
| Z_11OHAD | 0.12 | 5.69 |  | 180.95 | <0.001 |
| Z_11KT | 0.06 | 4.58 |  | 163.81 | <0.001 |
| Z_T | 0.66 | 8.91 |  | 109.33 | <0.001 |
| Z_21DOF | 0.37 | 1.86 |  | 8.13 | <0.001 |

| **Supplementary Table 2:** Comparison of baseline clinical characteristics in pubertal 21OHD patients | | | | |
| --- | --- | --- | --- | --- |
|  | poorly-controlled group  (n=8) | well-controlled group  (n=41) | Z/χ2 | P |
| Gender (male: female) | 5:3 | 16:25 | 1.51 | 0.26 |
| clinical phenotype (SW: SV: NC) | 5:2:1 | 12:19:10 | 2.83 | 0.29 |
| follow-up duration | 9.66 (4.16;12.36) | 6.50 (1.97;9.99) | 1.19 | 0.23 |
| Age (years) | 11.68 (8.75;12.60) | 10.96 (8.64;14.96) | 0.58 | 0.56 |
| Height SDS | 0.45 (-1.38;1.58) | 0.80 (-0.65;2.25) | 0.60 | 0.55 |
| Weight SDS | 1.90 (0.43;3.40) | 1.30 (0.55;2.40) | 0.95 | 0.34 |
| BMI-SDS | 1.80 (0.60;2.60) | 1.20 (0.60;2.20) | 0.83 | 0.41 |
| Hydrocortisone doses (mg/㎡) | 14.35 (8.13;18.07) | 15.38 (12.81;19.05) | 0.93 | 0.35 |
| Fludrocortisone dose (mg/d) | 0.05 (0.00;0.10) | 0.00 (0.00;0.04) | 1.67 | 0.09 |
| Note: Univariate analysis: The Mann–Whitney U test was used for quantitative data, and the χ² test for comparison of categorical data. | | | | |
|  | | | | |

| **Supplementary Table 3:** Androgen pathway activity and dominant pathway distribution in pubertal 21OHD patients | | | | |
| --- | --- | --- | --- | --- |
|  | poorly-controlled group  (n=8) | well-controlled group  (n=41) | Z/χ2 | P |
| **Robust Z-scores of** **different androgen pathways** | | | | |
| Z-Classical pathway | 6.53 (4.80;12.33) | 0.23 (-0.25; 0.76） | 4.44 | <0.001 |
| Z-11-Oxygenated pathway | 4.53 (3.87;6.35) | 0.23 (-0.53;0.93) | 4.30 | <0.001 |
| Z-Backdoor pathway | 4.02 (2.74;13.32) | 0.00 (-0.45;0.79) | 4.39 | <0.001 |
| Total robust Z-score | 15.80 (11.73; 31.48) | 0.83 (-0.96; 2.42) | 4.38 | <0.001 |
| **Relative weight of different** **androgen pathways** | | | | |
| Classical pathway (%) | 63.92 (45.07; 79.49) | 46.19 (5.43; 57.68) | 2.28 | 0.02 |
| 11-Oxygenated pathway (%) | 12.07 (0.00; 38.86) | 31.67 (0.00; 62.66) | 1.01 | 0.31 |
| Backdoor pathway (%) | 0.00 (0.00; 50.23) | 19.55 (0.00; 52.77) | 0.90 | 0.37 |
| **Distribution of dominant pathways** | | | | |
| classical pathway dominance (n, %) | 6 (75.00%) | 16 (39.02%) | 4.29 | 0.14 |
| 11-Oxygenated pathway dominance (n, %) | 0 | 13 (31.71%) |  |  |
| Backdoor pathway dominance (n, %) | 2 (25.00%） | 12 (29.27%) |  |  |
| **Pairwise Comparison of Androgen Pathway Relative Weights in the Poorly Controlled Group** | | | | |
| Classical vs 11-Oxygenated pathway (median, %) | 63.92 vs 12.07 | — | 2.52 (0.89) | 0.01 |
| Classical vs Backdoor pathway (median, %) | 63.92 vs 0.00 | — | 2.10 (0.74) | 0.04 |
| Backdoor vs 11-Oxygenated pathway (median, %) | 0.00 vs 12.07 | — | 0.14 | 0.89 |
| Note: Univariate analysis: The Mann–Whitney U test was used for quantitative data, and the χ² test for comparison of categorical data. Effect sizes are shown in parentheses after Z value | | | | |

| **Supplementary Table 4:** Correlation analysis of key enzyme conversion efficiencies across metabolic pathways in pubertal 21OHD patients | | | | | | | | |
| --- | --- | --- | --- | --- | --- | --- | --- | --- |
|  | C-E1 | C-E2 | C-Et | 11O-E3 | 11O-E4 | 11O-Et | Bd-E | 21DF-E |
| C-E1 | ­ | -0.79^*^ | 0.67 | -0.84^**^ | -0.71^*^ | -0.69 | 0.83^*^ | -0.33 |
| C-E2 | -0.26 | ­ | -0.14 | 0.81^*^ | 0.64 | 0.74^*^ | -0.69 | -0.02 |
| C-Et | 0.82^**^ | 0.17 | ­ | -0.35 | -0.55 | -0.36 | 0.64 | -0.45 |
| 11O-E3 | -0.41^**^ | 0.26 | -0.20 | ­ | 0.49 | 0.79^*^ | -0.63 | 0.26 |
| 11O-E4 | -0.45^**^ | 0.19 | -0.26 | 0.91^***^ | ­ | 0.81^*^ | -0.95^***^ | -0.19 |
| 11O-Et | -0.43^**^ | 0.23 | -0.23 | 0.97^***^ | 0.98^***^ | ­ | -0.20 | -0.05 |
| Bd-E | 0.92^***^ | -0.17 | 0.81*** | -0.18 | -0.25 | -0.20 | ­ | -0.09 |
| 21DOF-E | 0.70^***^ | -0.18 | 0.66*** | -0.19 | -0.29 | -0.23 | 0.75^***^ | ­ |
| Note: The lower left side of the table shows Spearman's correlation coefficients *r* for the well‑controlled group, and the upper right side shows Spearman's correlation coefficients *r* for the poorly controlled group; *P<0.05, **P<0.01, ***P<0.001; “—” indicates self‑correlation of the same indicator, which is not statistically significant | | | | | | | | |
